# Utility of a Machine-Guided Tool for Assessing Risk Behavior Associated With Contracting HIV in Three Sites in South Africa: Protocol for an In-Field Evaluation

**DOI:** 10.2196/30304

**Published:** 2021-12-02

**Authors:** Mohammed Majam, Mothepane Phatsoane, Keith Hanna, Charles Faul, Lovkesh Arora, Sarvesh Makthal, Akhil Kumar, Kashyap Jois, Samanta Tresha Lalla-Edward

**Affiliations:** 1 Ezintsha Faculty of Health Sciences University of Witswatersrand Johannesburg South Africa; 2 IPRD Solutions New York, NY United States

**Keywords:** machine learning, predictive risk, modeling, algorithm, HIV status, HIV, risk assessment, South Africa

## Abstract

**Background:**

Mobile technology has helped to advance health programs, and studies have shown that an automated risk prediction model can successfully be used to identify patients who exhibit a high probable risk of contracting human immunodeficiency virus (HIV)*.* A machine-guided tool is an algorithm that takes a set of subjective and objective answers from a simple questionnaire and computes an HIV risk assessment score.

**Objective:**

The primary objective of this study is to establish that machine learning can be used to develop machine-guided tools and give us a deeper statistical understanding of the correlation between certain behavioral patterns and HIV.

**Methods:**

In total, 200 HIV-negative adult individuals across three South African study sites each (two semirural and one urban) will be recruited. Study processes will include (1) completing a series of questions (demographic, sexual behavior and history, personal, lifestyle, and symptoms) on an application system, unaided (assistance will only be provided upon user request); (2) two HIV tests (one per study visit) being performed by a nurse/counselor according to South African national guidelines (to evaluate the prediction accuracy of the tool); and (3) communicating test results and completing a user experience survey questionnaire. The output metrics for this study will be computed by using the participants’ risk assessment scores as “predictions” and the test results as the “ground truth.” Analyses will be completed after visit 1 and then again after visit 2. All risk assessment scores will be used to calculate the reliability of the machine-guided tool.

**Results:**

Ethical approval was received from the University of Witwatersrand Human Research Ethics Committee (HREC; ethics reference no. 200312) on August 20, 2020. This study is ongoing. Data collection has commenced and is expected to be completed in the second half of 2021. We will report on the machine-guided tool’s performance and usability, together with user satisfaction and recommendations for improvement.

**Conclusions:**

Machine-guided risk assessment tools can provide a cost-effective alternative to large-scale HIV screening and help in providing targeted counseling and testing to prevent the spread of HIV.

**Trial Registration:**

South African National Clinical Trial Registry DOH-27-042021-679; https://sanctr.samrc.ac.za/TrialDisplay.aspx?TrialID=5545

**International Registered Report Identifier (IRRID):**

DERR1-10.2196/30304

## Introduction

A recent study by Nguyen et al [1] showed that albeit the current progress achieved by some African countries in the effort to work toward reaching the Joint United Nations Programme on HIV/AIDS (UNAIDS) targets of human immunodeficiency virus (HIV) testing, the prediction shows little chance in achieving this by 2030. None of the African countries analyzed shows a high probability of achieving the targets; however, only three countries still have a chance of attaining this, with a probability below 50%. This may suggest that countries heavily affected by HIV may require substantial efforts, such as increased support from global organizations, to make progress toward the UNAIDS targets. Additionally, there is a need to find and make more effective changes in the previous strategies for encouraging behavioral interventions to increase condom usage, and strengthen HIV programs in many of the most affected countries. HIV-screening programs therefore are largely invested in preventing HIV transmission by promoting regular testing and continuous development of innovative prevention methods and messages [2,3]. Mobile technology has played a role in advancing health programs [4,5], and studies have shown that an automated risk prediction model can successfully be used to identify patients who exhibit a high probable risk of contracting HIV [6-8]. One of these studies demonstrates that with the use of machine-guided risk assessments, the process of pre-exposure prophylaxis (PrEP) initiation can become more efficient and suitable.

Although identifying persons who are at high risk of contracting HIV should enable a more targeted and cost-effective approach for prevention programs, universal screening programs are costly and labor intensive and do not have as high a success rate in identifying high***-***risk individuals [6,9].

Supervised learning is used to train machine learning models by using patient traits as inputs. There are two broad categories of patient traits:

1. Generic patient traits, such as age, gender, disease history, and more
2. Disease-specific traits, such as sexual behavior in the context of HIV

In the supervised learning paradigm, we need to know the ground truth to train the model, which in this case is a medical outcome, such as a diagnostic test result. The relationship between patient traits and outcomes can be analyzed by measuring the performance of the model [10,11].

A risk assessment tool is a set of simple and easy*-*to*-*understand questions *that* are answered by the participant(s)*,* and based on the responses, a risk probability is calculated. The questions are related to demographics, sexual behavior, sexual history, personal behavior, lifestyle*,* and medical history. The questions are scientifically designed based on the HIV correlation data from published research and clinical trials. The tool makes use of scientific algorithms to determine the risk probability and *provides* the outcome as low risk*,* medium risk*,* or high risk. Diagnostic assistive assessment is performed on a mobile device by the patients themselves*,* thus eliminating external influences and hence providing more accurate information.

In 2019-2020, a feasibility study was conducted with over 1000 participants at the Wits Reproductive Health and HIV Institute’s (WRHI) HIV self-testing assessment and research (HSTAR) Africa site in Johannesburg, South Africa. This study sought to understand the correlation between HIV status and factors such as demographics, sexual behavior and history, personal behavior and lifestyle, and symptoms, individually.

Analysis of the data collected from this feasibility study showed correlations between HIV status and all the variables. The main inference made was that patients can be grouped based on multiple factors as follows:

- Primary features (demographics)
- Secondary features (lifestyle)
- Tertiary features (sexual behavior)
- Auxiliary features (symptoms)

**Figure 1:**
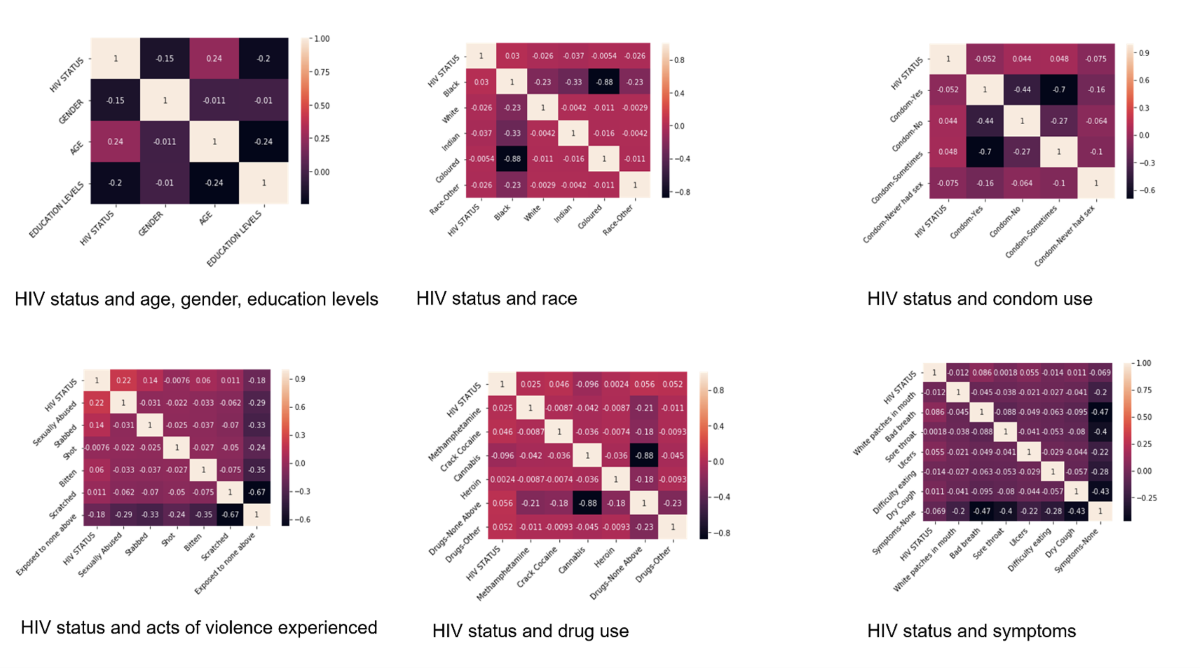
Examples of correlation between HIV status and selected features. Note: +1 shows a strong positive correlation, -–1 shows a strong negative correlation, and 0 shows no correlation between the variables. HIV: human immunodeficiency virus.

Examples of the detailed analysis are shown in Figure 1.

## Methods

### Objectives

The primary objective of this study is to assess the participants’ risk behavior using an automatic machine-guided tool and compare these results to HIV outcomes. The secondary objectives are:

- To evaluate the participants’ interaction with the tool in terms of effectiveness and efficiency, that is, successful/unsuccessful completion and difficulty using the tool
- To assess the ability of the participants to correctly comprehend and complete specific behavioral questions on a digital platform
- To understand user experience and satisfaction with the overall process and recommend improvements for machine-guided tool development

The study is registered on the South African National Clinical Trial Registry (www.sanctr.gov.za; DOH-27-042021-679) and was approved by the University of Witwatersrand Human Research Ethics Committee (HREC; ethics reference no. 200312) on August 20, 2020.

### Design

In this longitudinal, supervised learning study, the data collected will be split into a training set and a validation set and the data used to validate the model will not be used in the training of the model, which will eliminate the need for a control group. This study will build on the feasibility study described before. The major variable shift is from a clinic-based setting to an in-field setting, which includes rural and semirural sites.

During the evaluation, the untrained user will be assessed thoroughly for process success or difficulty by a silent, non-interacting nurse/counselor. Overall, the process will include (1) completing a series of behavioral questions on an application system without the assistance of trained staff (assistance will only be provided on user request); (2) HIV testing being performed by the trained nurse/counselor (for comparator data to the risk assessment scores); and (3) communicating test results and completing a survey questionnaire developed to collect data on user experience.

**Figure 2:**
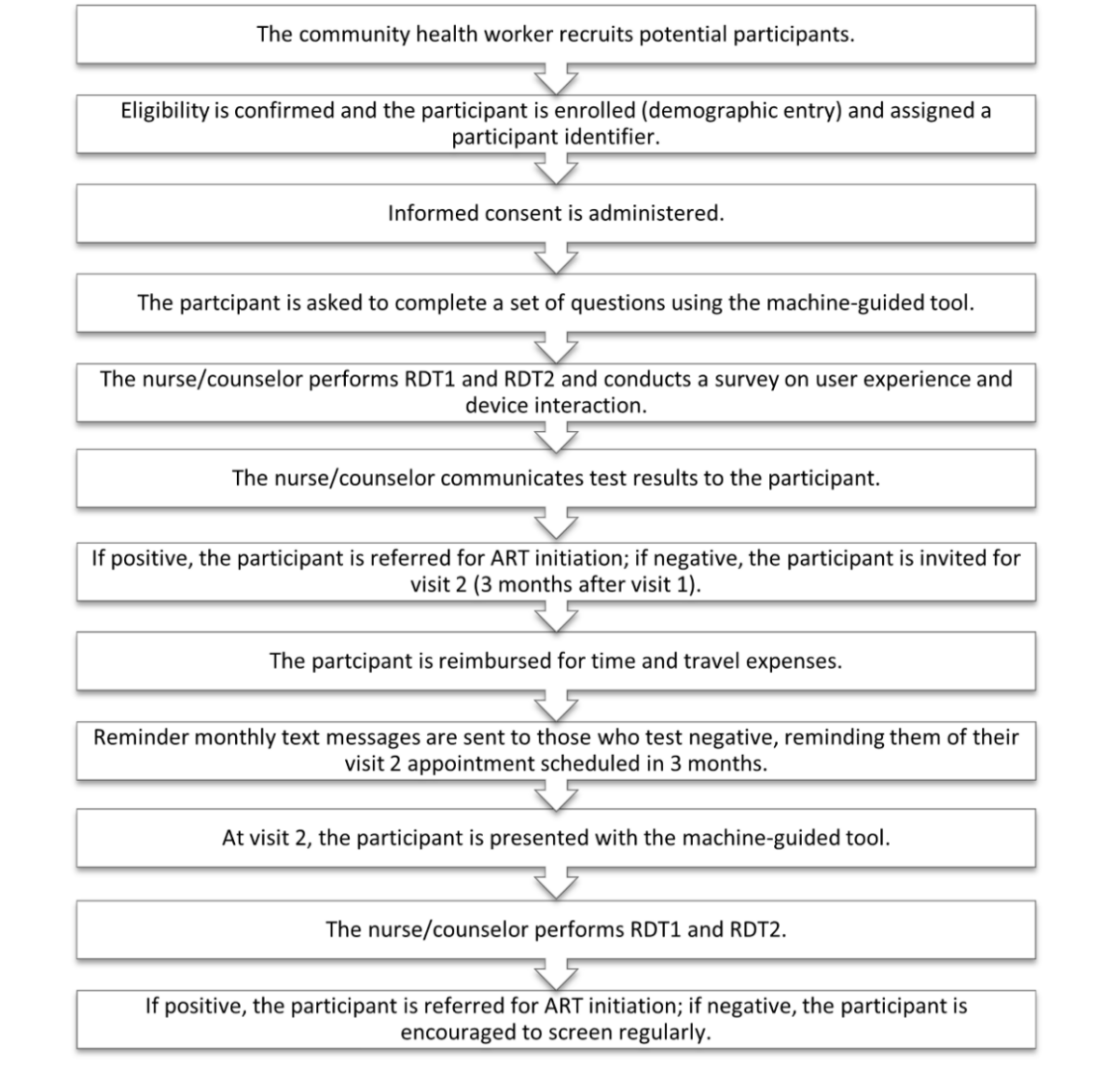
Flowchart of the study process from recruitment to confirmatory testing. ART: antiretroviral therapy; RDT: rapid diagnostic test.

All participants will have two rapid HIV tests performed. In the event of a positive HIV diagnosis, the participant will be referred for clinical treatment and care. If a participant tests negative, they will be found eligible for visit 2, which will occur 3 months after visit 1. At visit 2, the participant will follow the same process as that completed at visit 1 (Figure 2).

### Study Population

This is a general population study of adult males and females who are self-reported HIV negative or have an unknown HIV status in order to assess the capability, from device interaction, of identifying those who are potentially at high risk for contracting HIV. Recruitment will take place in different geographical regions using multiple recruitment methods, increasing the chances of the study population being representative of the general population.

### Study Site

Participants will be recruited from three districts situated in three provinces: (1) Tshwane, Gauteng; (2) Gert Sibande, Mpumalanga; and (3) Ugu, Kwa-Zulu Natal. The participating sites comply with all local government requirements for HIV testing and reporting. All sites will receive necessary approvals prior to enrolling participants.

### Sample Size

A purposive sample of 600 participants across all sites (200 per site) will be recruited for this study. In machine learning, the required sample size for a study has not developed into a clear-cut methodology and requires data to compare model performance to relative data size. Therefore, the initial feasibility study maximized on available funding to determine an initial sample size for method validation and to allow for the ability to use the initial sample to compare the performance of the model to the sample size. As seen in Figueroa et al [12], 80-560 samples were required to reach a root-mean-square error below 0.01. A sample size of 600 based on the observations of Figueroa et al [12] will facilitate comparison between model sample size and model performance in order to inform further investigations and machine learning model development. As the device is intended to be used by both males and females from the general population, this assessment aims to recruit as close to a 50% breakdown per gender as possible.

### Inclusion Criteria

This study is open to individuals 18 years of age or older who meet the inclusion criteria. No potential participant will be excluded because of race, gender, ethnicity, or sexual orientation.

People volunteering to be enrolled in this study must meet the following criteria:

- Understands and signs the written informed consent form
- Is able to complete the required testing on the allocated testing day(s)
- Agrees to provide an accurate medical history and the required specimens for two fingerprick blood tests
- Is able to speak and read English
- Is ≥18 years of age
- Is willing to provide the required information for the algorithms
- Is HIV negative or has an unknown HIV status

### Exclusion Criteria

Participants meeting any of the following criteria will be excluded from the study:

- Are known to be HIV positive
- Have received any experimental HIV vaccine
- Are currently on a PrEP regimen or any HIV treatment
- Cannot provide legal identification for age verification
- Have any condition that, in the opinion of the facilitator, would make them unsuitable or unsafe for enrollment; interfere with the completion of the assessment, consent form, and questionnaire; or bias the outcome, for example, being unable to see/read by forgetting to bring reading glasses, being intoxicated, or having acute illness

### Blinding of Results

This will be a blinded study. Participants will not be aware of their risk assessment scores. There will be no randomization of participants, as all participants will be required to interact with the machine-guided tool. This study will make use of supervised learning in which the data set will be collected and then used to train and test the machine learning model that has been created. The data will be collected in a specific area, and selection will be random. The data, however, will be split into training and validation data using a randomized split algorithm commonly found in statistical and data processing languages such as R and Python.

### Recruitment

**Figure 3:**
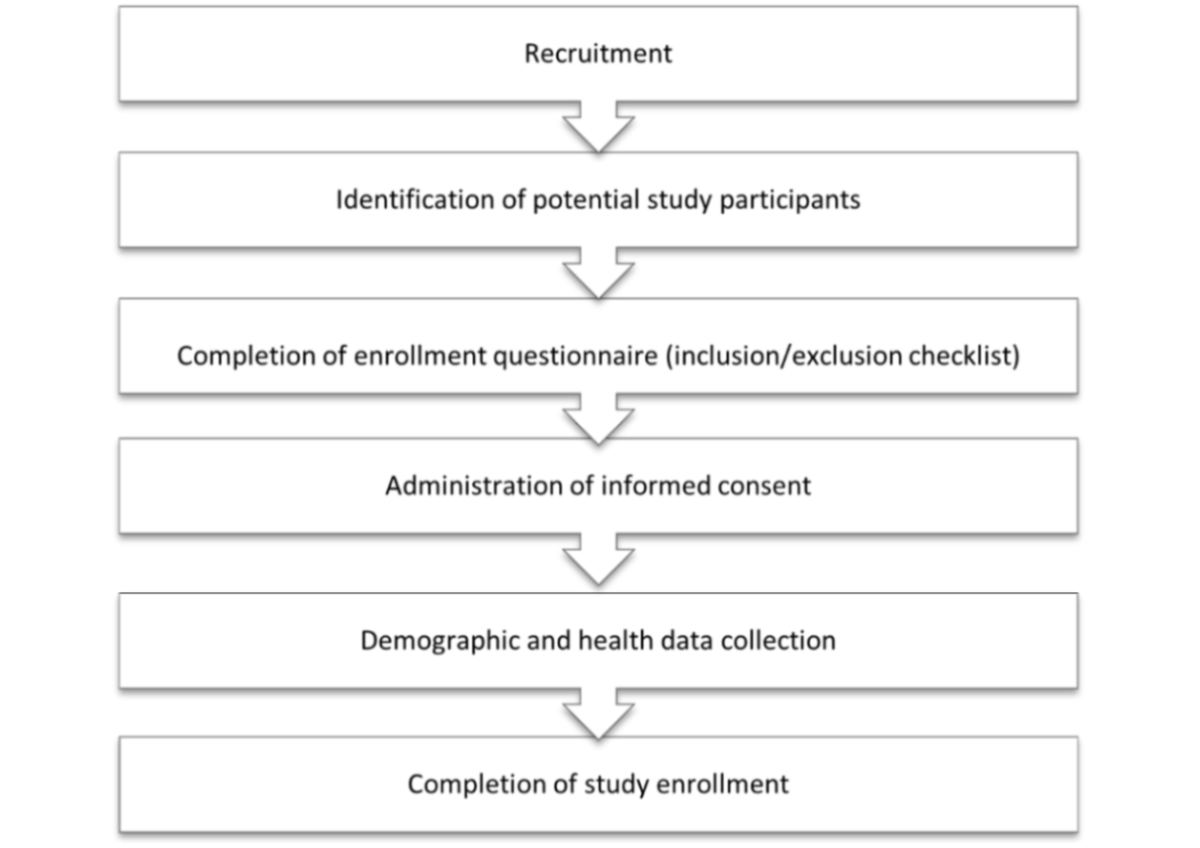
Flowchart of participant recruitment and study enrollment.

Participant selection will be recruitment based related to clearly defined inclusion and exclusion criteria described before. Selected participants will be required to sign an informed consent form. Project staff will be required to fill data collection forms and study questionnaires that include participant demographic information about age, education, language preference, and a brief medical history. Participant recruitment follows a serial process, as shown in Figure 3.

*Community-based recruitment*: Participants will be recruited by community health workers stationed at a mobile testing unit using regulatory body–approved study-designed recruitment materials. Additional recruitment methods will be used, such as outreach activities within the community and appearance at community events and local malls.

*Word-of-mouth recruitment*: Participants will be encouraged to tell others about the study. Once potential participants have been identified through the recruitment modes, they will be approached and informed about the study and the role they will play in the study procedure. The objectives, rationale, eligibility requirements, and procedures of the study will be explained to the participants and will highlight that participation is purely voluntary. The risks and benefits of participation in the study and the rights of participants in the study will also be discussed. The participants will be encouraged to ask questions to ascertain their level of understanding and asked to take flyers home with them to inform other potential participants. Potential participants who meet inclusion criteria will be scheduled and directed to the testing site.

### Enrollment Questionnaire: Inclusion/Exclusion Checklist

An enrollment questionnaire will be administered to establish successful qualification for enrollment. The questionnaire will clearly outline the inclusion and exclusion criteria. If participants satisfy all the inclusion criteria and none of the exclusion criteria, eligibility for enrollment will be confirmed. Enrollment questionnaires will be available in English and will capture information such as participant identification, name and surname, date of birth, nationality, employment status, and recruitment site. Information such as participant initials, date of birth, gender, date of enrollment, and reason for exclusion will be captured on an enrollment log. Once a participant has qualified for enrollment and has signed the informed consent form, the participant will be assigned a unique identification number. Volunteers who do not satisfy inclusion criteria will be documented and reported as exclusions. Further, the reason for enrollment failure will also be recorded.

### Selection and Withdrawal of Study Participants

Participants may voluntarily withdraw participation at any time. In this case, the principal investigator (PI) will acquire all study-related information and the reason for withdrawal will be documented. The information and data collected prior to participant withdrawal from the study may be used for research by the PI or sponsor. This information may be used without identifying any personal information as agreed upon in the informed consent form unless the participant provides a written request prohibiting/limiting the use of their study data.

The PI may decide to limit or withdraw participation of any participant at any point in the study. Further, the sponsor may close the study prematurely for any reasons, including administrative decisions.

### Informed Consent

Prior to any research processes taking place, potential participants will be provided with information about the study, enabling an informed decision regarding further participation. If the participant voluntarily accepts participation, a regulatory body–approved informed consent form will be administered. The form will contain general information about the study and specific information regarding sample collection and all other study procedures.

Potential participants will be encouraged to ask questions to ensure the entire process is clearly understood. Prospective participants will also be informed that they may reject the specimen collection procedure and withdraw from the study at any time. Participants must agree to and submit their informed consent forms (along with demographic data; see below) prior to formal enrollment in the study.

Informed consent will be offered after verbal explanation of the study procedures in English by designated staff. If a participant is found to be unable to read or write, no further consent procedures will be undertaken.

### Demographic and Health Data Collection

All enrolled participants will have the following demographic data captured:

- Initials
- Date of birth
- Age
- Gender
- Nationality

The participant background information captured will include:

- Employment status (employed/unemployed)
- Years of schooling and level (≤grade 7 primary schooling level/≥grade 8 primary schooling to ≤matric level/≥Technikon, university, and university plus)
- Reading/writing impairment
- Language preference
- HIV status (if known)
- Approximate date of last HIV test

Participants may choose to report their medical health history, which will be documented and archived for statistical review. Information will include:

- Self-reported HIV status:
- Unknown status/never been tested
- Negative status
- Self-reported medical conditions:
- Diabetes
- Hypertension
- Visual impairment
- Pregnancy
- Other

### Testing Procedures

#### Subject Tool Evaluation

After the consenting procedures, enrollment questionnaire, and demographic data have been collected, each participant will be invited into a private area (tent, gazebo setting) and introduced to the clinical staff (nurse/counselor), who will describe the study process. The digital tool interaction is to be completed under the direct observation of the study staff. The nurse/counselor will verbally guide the participant as follows: “This is the part of the study in which you will be asked to use the study product. With this product, you can begin to answer the questions yourself. I will be available to answer any questions or explain further if you require assistance, but would prefer that you try and use the tool on your own. When you are finished, you can let me know and we will proceed to testing.”

**Figure 4:**
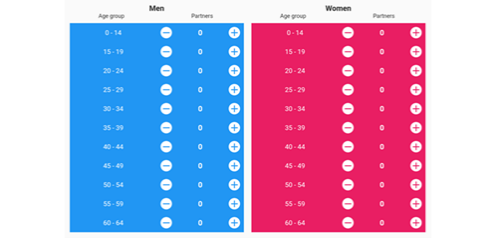
Number of partners and age groups selection box.

Each participant will be presented with a tablet that has the application installed and will be requested to complete the set of questions listed below:

1. Please select your gender.
  Male
  Female
  Transgender
2. Date of birth:
3. Please select your race.
  Black
  White
  Indian
  Colored
  Other (please specify)
4. What is your education level?
  Primary school
  High school
  Diploma or certificate
  Tertiary
  None of the above
5. What is your occupation type?
  Informal (different employers, depending on job) How long working away from home (remote)? 1 week per month 2 weeks per month 3 weeks per month 1 month or longer Working in the same city/area where you live (local)
  Formal (permanently employed and same boss) How long working away from home (remote)? 1 week per month 2 weeks per month 3 weeks per month 1 month or longer Working in the same city/area where you live (local)
  Unemployed (no job)
6. When was the last time you tested for HIV?
  0-3 months
  3-12 months
  More than 12 months
  Never tested
7. Do you use a condom during sex?
  Yes
  No
  Sometimes
  I have never had sex.
8. Please select the gender and age of all sexual partners with whom you had sex in the past 3 months.
  Number of partners and age groups (Figure 4)
  No sex in the past 3 months
9. Which of the following sexual activities have you performed in the past 6 months?
  Anal receptive (you receive penis in anus)
  Anal insertive (you insert penis in partner’s anus)
  Vaginal receptive (you receive penis in vagina)
  Vaginal insertive (you insert penis in partner’s vagina)
  Oral receptive (your partner sucks your vagina/penis)
  Oral insertive (you suck partner’s vagina/penis)
  None of the above
10. Have you gone through any of the following in the past 6 months?
  Sexually abused
  Stabbed
  Bitten
  Scratched
  None of the above
11. Have you experienced/noticed any or many of the following?
  Weight loss of more than 5 kg over 3 months
  Persistent cold and flu in the past 6 months
  Persistent diarrhea and fatigue in the past 6 months
  Recurring night sweats in the past few months
  White patches in mouth
  Bad breath
  Sore throat
  Ulcers
  Difficulty in eating
  Dry cough
  Wet cough for more than 3 weeks in the past 1 year
  Blood in cough in the past 1 year
  Persistent fever or chills for no known reasons in the past 1 year
  Persistent shortness of breath or chest pain in the past 1 year
12. Have you experienced/noticed any or many of the following?
  Genital warts (small bumps on genitals)
  Genital herpes (genital pains or sore genitals and open sores on genitals)
  Gonorrhea (bacterial infection: white or yellow liquid visible in genital region)
  Syphilis (rash on the body or painless sore on genitals, rectum, or mouth)
  Gonococcal (painful urination and abnormal discharge from penis or vagina)
  None of the above

A trained nurse/counselor will proceed to perform two rapid HIV tests and communicate the results to the participant. If the results are HIV positive, the participant will be counseled and encouraged to commence antiretroviral therapy at a clinic of their choice. If the results are HIV negative, the participant will be eligible for visit 2 (3 months after visit 1) and will be issued with an appointment card. If the results are discordant, the nurse/counselor will be required to repeat testing. Additionally, the participant will be sent visit 2 appointment reminders through text messages monthly.

#### Confirmatory Procedures

HIV status will be confirmed using the rapid diagnostic kits in-use (First Response HIV 1-2-0 and Alere DetermineHIV-1/2) at the testing site. If the confirmatory rapid test is HIV positive, the participant will be referred to a clinic of their choice for treatment and care.

In the event the second rapid test shows a negative result, the HIV status will be considered indeterminate, and all tests will be repeated (Textbox 1) [13].

**Interpreting test results. RDT: rapid diagnosis test.**

RDT1 (positive) + RDT2 (positive) = positive

RDT1 (negative) + RDT2 (negative) = negative

RDT1 (negative) + RDT2 (positive) = indeterminate (repeat test)

RDT1 (positive) + RDT2 (negative) = indeterminate (repeat test)

### Data Analysis

The study endpoints for this trial are concerned with the proportion for concordance of the participants’ risk assessment score with the confirmatory test results.

An interim analysis of the results will be reviewed after visit 1 has been completed. All the recorded risk behavioral scores will be analyzed for any correlation between HIV testing outcomes.

In addition, the analysis plan will include an evaluation performed at visit 2, identifying cases where an initial nonreactive test (at visit 1) has resulted in a change to a reactive test result (at visit 2).

#### Machine Learning Algorithm

The first phase of this machine-guided diagnostic study will be used to compare potential supervised learning algorithms to determine which algorithm is the most robust in variable variation. The algorithms that will be compared are K-nearest neighbor, logistic regression, neural network, random forest, support vector machine, and XGBoost. These models will be compared using a confusion matrix by comparing sensitivity, specificity, accuracy, precision, and negative predictive value. When comparing models, the relative risk of a false negative versus a false positive will be taken into consideration to minimize the risk of potentially high-risk patients not being flagged for further screening.

#### Error and Failure Rates

Critical errors occur when participants make operational errors during interaction with the machine-guided tool. Project staff will aid participants, if needed, to ensure accurate completion of all behavioral questions that contribute directly to risk assessment scores.

The failure rate (cases where the risk score is high and the test result remains negative for both visits) will be identified and reported as the number or a percentage of failed cases of the total number of cases completed in the study.

It should be noted that failure due to critical errors (participants not being able to successfully complete the digital assessment) will not be included in the analysis.

The type of behavioral questions and the weighted score for each will be monitored and evaluated for continuous improvement and refinement purposes.

#### Missing Data

The study will use electronic data capture for each participant, which requires that each question be completed before moving on to the next. This will eliminate the possibility of missing data during the data collection section of the study. Any participant record with missing results for a HIV test will be removed from the study.

#### Sensitivity Analysis

**Figure 5:**
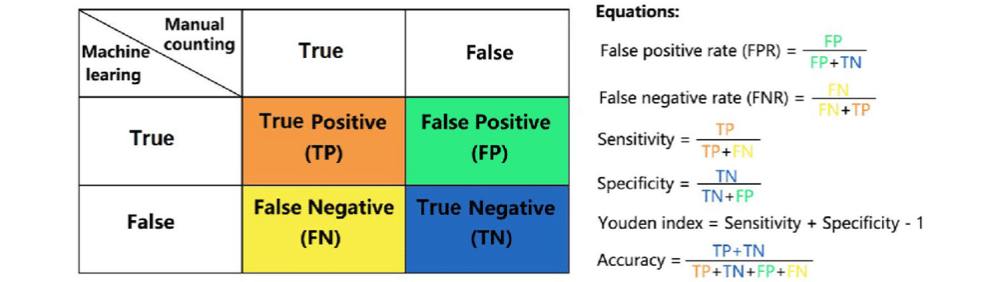
Confusion matrix with classification metrics [14].

The performance of the model will be analyzed using the validation data set from the data split using a confusion matrix (Figure 5) that will allow for the calculation of sensitivity, specificity, and accuracy.

#### Interaction Between Variables

A bivariate analysis will be performed for each variable to investigate the relationship between the respective variable and HIV status. This will then further be used in feature engineering.

#### Post Hoc Analysis

In machine learning, post hoc analysis is not commonly performed, and a comparison of model performance through the analysis of a confusion matrix and confidence curves offers an alternative to conventional omnibus tests [15].

#### Skewed Data

The participants enrolled in this study who are either HIV negative or of unknown HIV status will cause a lower HIV prevalence in the sample data set. However, this will be done so as to remove the possibility of participants who are HIV positive creating a bias in the supervised learning, as these people may have altered behavior due to being HIV positive, which may result in risk reduction behavior, reducing the sensitivity and accuracy of the model. The skewed data set will be accounted for in the second phase of the study, where participants will perform two visits, including a follow-up HIV test, which will allow for the analysis of a comparison to the predictive ability of the machine learning model created in phase 1.

### Secondary Objectives

The usability of the tool will be measured through direct observation. The study staff will complete a checklist and note whether each participant was *successful* or *unsuccessful* in using the tool. They will also note whether the participant was able to complete the behavioral questions via electronic data collection.

User satisfaction will be recorded by a study team member via a series of yes/no questions to obtain an overall satisfaction score. Additionally, recommendations will be captured as free text and coded during analysis.

### Data Management

Data management will be undertaken by the HSTAR staff. Data will be collected on paper (questionnaires, informed consent forms) and on tablets (demographic data collection) and will be transmitted daily to the research site data staff. A data capturer will enter the paper-based data into an in-house-developed electronic database within 2 days. Data quality control will be managed daily by the project team.

#### Confidentiality

All documents, reports, and other records will be identified in a manner designed to maintain participant confidentiality. All records will be kept in a secure storage area with limited access. Clinical information will not be released without the written permission of the participant, except as necessary for monitoring and auditing by regulatory authorities.

The PI or designee and the study team may not disclose or use for any purpose other than performance of the study, any data, record, or other unpublished confidential information disclosed to those individuals for the purpose of the study. All computers are password-protected, and records can only be accessed by permitted study staff.

#### Record Retention

All correspondence relating to this study will be kept in appropriate study folders. Records of participants, source documents, and questionnaires pertaining to the study will be kept on file. Essential documents will be retained until at least 2 years after the last approval of a marketing application in an International Conference on Harmonization (ICH) region and until there are no pending or contemplated marketing applications in an ICH region or at least 2 years have elapsed since the formal discontinuation of product development. These documents will be retained for a longer period, if required by the applicable regulatory requirements. If an investigator moves, withdraws from the study, or retires, the responsibility for maintaining the records will be transferred to another person who is willing to accept the responsibility.

#### Data Reporting

Data reporting will be performed by the program manager and study consultants. A research report will describe the study area, study population, and execution of the research and present the study results. The report will present both qualitative and quantitative discussions on the outcomes of the study.

Biannual progress reports will be submitted to the University of the Witwatersrand Human Research Ethics Committee (HREC) for the duration of the study. Annual recertification will be obtained from the HREC. Upon completion or premature termination of the study, the PI will provide the HREC with a summary of the study’s outcome and any other regulatory authorities with any reports required.

### Other Study Procedures

#### Investigator Documentation

Prior to study commencement, the PI will be asked to comply with ICH E6(R1) 8.2 by providing the following essential documents, including but not limited to:

- An original signed investigator agreement page of the protocol.
- An HREC-approved informed consent form, samples of site advertisements for recruitment for this study, and any other written information regarding this study that will be provided to the participants.
- HREC approval.
- Curriculum vitae of the PI and each investigator. They will be signed and dated by each investigator at study start-up, indicating that they are accurate and current.

#### Quality Control and Study Monitoring

Quality control of the study will be performed following the standard operating procedures developed for the study and those generic to the implementing organization. The project manager will assume overall responsibility for ensuring all procedures are adhered to and quality controlled.

A study monitor will be contracted. The study monitor will have the obligation to follow the study closely. In doing so, the study monitor will visit the study facility at periodic intervals, in addition to maintaining necessary telephonic and email contact. They will maintain current personal knowledge of the study through observation, review of study records and source documentation, and discussion of the conduct of the study with the PI and study staff.

All aspects of the study will be carefully monitored for compliance with applicable government regulation with respect to current ICH good clinical practice (GCP) guidelines and current standard operating procedures.

#### Protocol Amendments

Protocol amendments will be prepared by the PI. These amendments will be submitted in writing to the HREC for approval prior to participants being enrolled into the amended protocol, except where it is necessary to eliminate an immediate hazard to participants or where the changes involve only logistical or administrative aspects of the clinical study. This will be fully documented.

Examples of amendments requiring approval are:

- A significant change in the study design
- An addition or deletion of a test procedure for safety monitoring

The requirements for approval will in no way prevent any immediate action from being taken by the investigator or the sponsor in the interests of preserving the safety of all participants included in the study. If an immediate change to the protocol is deemed necessary by the investigator and is implemented by them for safety reasons, the PI will be notified and the HREC will be informed within 10 working days.

#### Protocol Violations and Deviations

A deviation from the protocol is an unintended or unanticipated departure from the procedures or processes approved by the HREC and agreed to by the PI or designee. Deviations usually have an impact on individual participants or a small group of participants and do not involve inclusion, exclusion, or primary endpoint criteria.

A protocol violation occurs when there is non-adherence to the protocol that results in a significant additional risk to the participant, when the participant or PI or designee fails to adhere to significant protocol requirements (inclusion and exclusion criteria) and the participant is enrolled without prior approval, or when there is non-adherence to regulations or some ICH GCP guidelines.

The PI or designee will document and explain in the participant’s source documentation any deviation from the approved protocol. The PI or designee may implement a deviation from or a change of protocol to eliminate an immediate hazard to trial participants without prior HREC approval. As soon as possible after such an occurrence, the implemented deviation or change, the reasons for it, and any proposed protocol amendments will be submitted to the HREC for review and approval.

Protocol violations and deviations will be documented by the clinical monitor throughout the course of monitoring visits. The PI or designee will be notified in writing by the monitor of violations and deviations. The HREC will be notified of all protocol violations and deviations in a timely manner.

#### Adverse Events and Adverse Device Event Reporting

Adverse events will be captured, and line listings provided. Any serious adverse event or adverse device event that may occur will be reported to the PI. Depending on the severity of the adverse event, the PI is obliged to report the event to the HREC. All serious adverse events will be reported by the PI.

#### Inspection of Records

The PI or designee and institutions involved in the study will permit study-related monitoring, audits, HREC reviews, and regulatory inspections by providing direct access to all study records.

#### Study Termination

Although there is every intention to complete the study, the implementing organization reserves the right to discontinue the study at any time for clinical or administrative reasons. The end of the study is defined as the date on which the last participant completes the last visit.

#### Dissemination

De-identified results will be disseminated through community engagements, study reports, and peer-reviewed publications and conference presentations. All outputs will be produced in compliance with donor requirements.

## Results

This study received ethical approval from the University of Witwatersrand HREC (ethics reference no. 200312) on August 20, 2020, and has been funded by the Bill & Melinda Gates Foundation (OPP 1204282). As an ongoing study, data collection has commenced and is expected to be completed in the second half of 2021. At the end of this study, we will be able to report on the performance and usability of the machine-guided tool, that is, the accuracy of the tool to identify people who are at high risk of contracting HIV and how easy participants find it to successfully interact with the digital platform. Lastly, the results of this study will report on user satisfaction and their recommendations for improvement. The results will be disseminated by the first quarter of 2022.

## Discussion

### Importance of Principal Findings

To the best of our knowledge, this would be the first evaluative report on a machine-guided tool for assessing the risk behavior associated with HIV acquisition in the South African context. South Africa has a high burden of HIV, and less than 90% of the population knows its HIV status [16]. Despite the large financial investment in the country, prevention of HIV infection remains a challenge [17]. In evolving economic landscapes and contexts that necessitate employment of innovative approaches to addressing HIV, machine guidance has the potential to offer a cost-effective, focused solution to identify priority populations for HIV prevention and treatment. Our study will provide evidence for this approach as a possible solution to tackling HIV risk and identification.

Machine guidance and artificial intelligence have proven useful in the health sector and are being used in many branches of medicine, including gastroenterology and hepatology [18], oncology [19,20], and infectious diseases [21,22]. For HIV, the potential benefits of engaging in machine-guided identification include immediate and private HIV test results, which may encourage frequent testing of individuals in high-risk groups who would otherwise refrain from testing due to the unknown risk factors associated with susceptibility to acquiring HIV. Although many health ministries make available free testing for HIV in a clinical setting, this machine-guided identification removes the need for spending time visiting a clinic and waiting for results, which is particularly critical where public testing facilities require long-distance traveling.

Nguyen et al [23] highlight the importance of understanding high-risk behaviors (eg, multiple concurrent partnerships, unprotected sex, and needle sharing) and demographic characteristics of people newly diagnosed with HIV/acquired immunodeficiency syndrome (AIDS). It is crucial for health programmers to consider this information as they design strategies for preventing HIV acquisition and transmission. With an earlier diagnosis, individuals are empowered to identify and modify their high-risk behaviors, potentially reducing the number of new HIV infections. This empowerment of consumers is not just limited to modifying lifestyle decisions but also involves being proactive in their health care decisions, which include adherence to clinic appointments and HIV treatment adherence (crucial to the prevention of viral rebound, treatment resistance, and the success of HIV management programs) [24,25].

The risk probability of contracting HIV may positively impact the effectiveness of universal testing and treatment by directing limited testing/treatment resources to the highest-risk groups, the number of people knowing their HIV status, the number of people linked to services, decision making and follow-up costs, reduction in duplication of tests, identification of high-risk self-harm cases, and PrEP initiation.

### Conclusion

Machine-guided risk assessment tools can provide a cost-effective alternative to large-scale HIV screening. This will be beneficial in providing directed counseling and testing to prevent the spread of HIV.

## Abbreviations

AIDS: acquired immunodeficiency syndrome
ART: antiretroviral therapy
GCP: good clinical practice
HIV: human immunodeficiency virus
HREC: Human Research Ethics Committee
HSTAR: HIV self-testing assessment and research
ICH: International Conference on Harmonization
PI: principal investigator
PrEP: pre-exposure prophylaxis
RDT: rapid diagnostic test
UNAIDS: Joint United Nations Programme on HIV/AIDS
